# Conductive Gels for Energy Storage, Conversion, and Generation: Materials Design Strategies, Properties, and Applications

**DOI:** 10.3390/ma17102268

**Published:** 2024-05-11

**Authors:** Gazi A. K. M. Rafiqul Bari, Jae-Ho Jeong, Hasi Rani Barai

**Affiliations:** 1 School of Mechanical Smart and Industrial Engineering, Gachon University, 1342 Seongnam-daero, Sujeong-gu, Seongnam-si 13120, Gyeonggi-do, Republic of Korea; grafiqulbari@gachon.ac.kr; 2 School of Mechanical and IT Engineering, Yeungnam University, Gyeongsan 38541, Gyeongbuk, Republic of Korea

**Keywords:** gels, energy, polymers, conductive gels, hydrogels, flexibility, wearable devices

## Abstract

Gel-based materials have garnered significant interest in recent years, primarily due to their remarkable structural flexibility, ease of modulation, and cost-effective synthesis methodologies. Specifically, polymer-based conductive gels, characterized by their unique conjugated structures incorporating both localized sigma and pi bonds, have emerged as materials of choice for a wide range of applications. These gels demonstrate an exceptional integration of solid and liquid phases within a three-dimensional matrix, further enhanced by the incorporation of conductive nanofillers. This unique composition endows them with a versatility that finds application across a diverse array of fields, including wearable energy devices, health monitoring systems, robotics, and devices designed for interactive human-body integration. The multifunctional nature of gel materials is evidenced by their inherent stretchability, self-healing capabilities, and conductivity (both ionic and electrical), alongside their multidimensional properties. However, the integration of these multidimensional properties into a single gel material, tailored to meet specific mechanical and chemical requirements across various applications, presents a significant challenge. This review aims to shed light on the current advancements in gel materials, with a particular focus on their application in various devices. Additionally, it critically assesses the limitations inherent in current material design strategies and proposes potential avenues for future research, particularly in the realm of conductive gels for energy applications.

## 1. Introduction

The increasing global demand for energy materials, crucial for energy storage and conversion across various applications, underscores the pivotal role of gel-based materials. Gel-based materials present a promising alternative due to their versatile utility [1]. In recent years, these materials have garnered significant attention owing to their remarkable structural flexibility, facile modulation, and cost-effective synthesis methodologies [2,3,4]. The three-dimensional scaffold of gel materials provides a large surface area hosting active sites with adequate composition, structural integration, chemical stability, and eco-friendliness [5,6,7]. Gel materials play multidimensional roles as electrolytes, electrodes, and binders, favoring intrinsic stretchability, bending ability, and flexibility, making them suitable for soft electronic energy devices [8,9]. Notably, polymer-based conductive gels, characterized by their unique conjugated structures integrating both localized sigma and π-bonds, have emerged as preferred materials across a broad spectrum of applications. These gels exhibit exceptional integration of solid and liquid phases within a three-dimensional matrix, further enhanced by the inclusion of conductive nanofillers. Such compositional uniqueness confers upon them versatility extending across various domains, including wearable energy devices, health monitoring systems, robotics, interactive human-body integration devices, and electrochemical energy conversion systems such as metal–air batteries, fuel cells, and water-splitting electrolyzers [10].

Evident from their inherent stretchability, self-healing capabilities, and both ionic and electrical conductivity, alongside their multidimensional attributes, gel materials manifest multifunctionality [11,12,13]. Additionally, the three-dimensional structure of gel materials effectively accommodates volume expansions and facilitates effective ion movement during catalytic operations. However, the effective integration of these multidimensional properties into a singular gel material, tailored to meet diverse mechanical and chemical requirements across applications, remains a formidable challenge. Challenges related to conductive hydrogels, such as multifunctionalities encompassing stretchability, anti-freezing properties, self-healing capabilities, self-adhesive properties, and electrochemical properties, necessitate a balance among different components for enhancement [14,15]. Challenges related to drying, freezing properties, and achieving self-healing properties are essential to maintaining integrity. Constructing conductive gels relies on incorporating conductive polymers, metals, carbon-based materials, and ionic salts into 3D networks, where maintaining stable operation poses challenges due to phase separation between conductive additives and polymer networks, leading to mechanical and performance deficiencies. Given the sensitivity of gel materials to environmental conditions, it is paramount to withstand harsh conditions, including extreme cold or hot environments, to extend service periods [16,17,18,19]. Furthermore, validating gel materials in response to stimuli such as temperature, electric fields, magnetic fields, light, pressure, sound, pH, solvent composition, ionic strength, and molecular species is challenging. Biocompatibility is crucial for wearable applications, especially when in close contact with the skin [20,21,22].

The preparation of electronic conductive gels involves the incorporation of various conductive fillers, including metals, carbon-based derivatives, and conductive polymers. However, achieving compatibility and strong interfacial interactions between gels and metals poses a significant challenge, as the lack thereof may result in microstate phase separation, thereby impeding the operational feasibility of the conductive gel. Modification of metals, such as shaping or sizing into nanorods, nanowires, nanoparticles, or even employing liquid metals like Ga, holds promise for enhancing conductivity or performance [23,24,25]. Alternatively, the utilization of 2D inorganic materials such as transition metal carbides or carbonitrides in energy storage and catalysis for conductive gels offers advantages such as high strength, self-healing, and thermal conductivity properties, attributable to workable crosslinking points [26]. While carbon nanotube (CNT)-based fillers provide nano reinforcement and intrinsic conductivity, fabricating gels with them presents challenges due to the hydrophobic nature of CNTs, which may compromise mechanical strength. Similarly, conductive polymers represent potential candidates for fabricating conductive gels; however, they lag due to poor strength, low conductivity, and the rigidity of conjugated polymer chains [27]. Strategies such as doping with phytic acid, which enhances conductivity in polyaniline (PANI)/polypyrrole (PPy) gels, show promise. Moreover, incorporating conductive monomers into gels to trigger in situ polymerization holds the potential for decreasing agglomeration, intensifying conductivity, and improving mechanical properties, as demonstrated by the use of poly(3,4-ethylenedioxythiophene):poly (styrene sulfonate) (PEDOT:PSS) in PPy/PANI systems [28,29]. Ionic conductive gels incorporate various traditional electronically conductive materials, enabling the unhindered movement of ions through a facilitated porous structure. Also, the design facilitates the conduction of multiple ions, such as Li^+^ and Na^+^ ions, as well as ions from clay, within a single hydrogel system, harnessing both conductivity and mechanical robustness [30,31,32]. The anticipated multifunctional properties of these gels include mechanical resilience, mechanosensitivity, freeze resistance, self-healing capabilities, self-adhesion, and moisture retention, surpassing the singular functionality of mere conductive gels.

This review aims to elucidate recent advancements in gel materials, with a particular emphasis on their utilization in diverse devices. Moreover, it critically evaluates the intrinsic limitations of prevailing material design approaches and delineates potential avenues for future research, particularly in the domain of conductive gels for energy applications.

## 2. Conductive Gel Materials Formation, Classifications, and Fabrications

**Figure 1:**
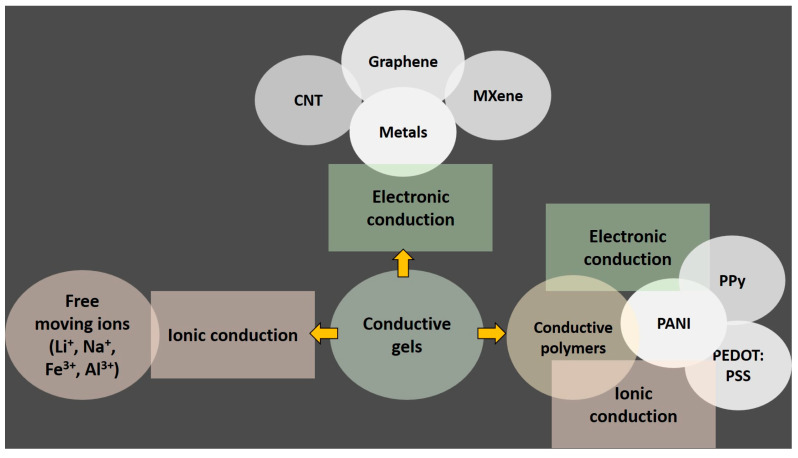
Conductive gel design strategies.

Gel materials consist of three-dimensional polymer networks or non-fluid colloids saturated with liquid. Their porous architecture, abundant with defects, presents numerous opportunities for mass transfer, allowing for the customization of composition to meet specific application needs. Conductive gels, in particular, hold great promise for a wide range of multidimensional applications. The performance and synthesis of conductive gels typically revolve around two strategies: electronic conduction and ionic conduction. Conductive gel can be created by adding conductive materials to the gel substrate or by directly incorporating conductive substances (Figure 1). Carbon materials such as graphene and carbon nanotubes contain π-electrons, which facilitate π–π interactions to form conductive networks. MXene, possessing conductivity similar to metals, also forms hydrogen bonds with conducting polymers, thereby ensuring the formation of conductive pathways [33,34].

**Figure 2:**
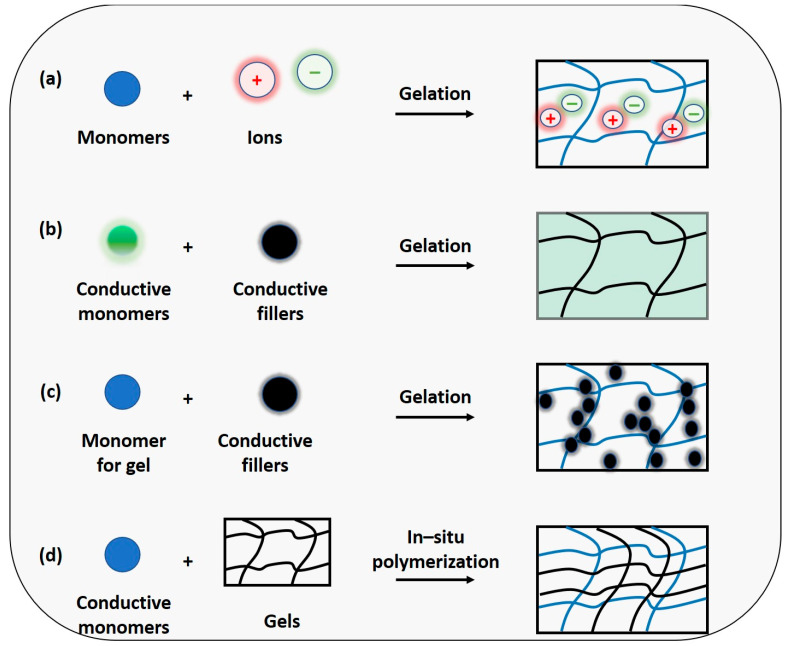
The conductive hydrogel design principle is based on (**a**) conductive medium hydrogel, ionic conductive hydrogel, (**b**) conductive network hydrogel, electronic conductive hydrogel, (**c**) conductive medium hydrogel, electronic conductive hydrogel, and (**d**) conductive network hydrogel, electronic conductive hydrogel.

Conductive hydrogels can be obtained through two approaches. One method involves the intrinsic incorporation of metal matrices (such as Au, Cu, Ag, etc.) in the form of nanowires/nanoparticles, or the incorporation of a conductive matrix (graphene, carbon nanotubes, MXene). Alternatively, another approach involves conductive polymers such as PANI, PEDOT:PSS, and PPy [35,36]. Another approach entails achieving ionic conductivity by adding salts or charged materials to the hydrogel network (Figure 2). Metal-based nanoparticles or fillers provide excellent electrical conductivity for efficient electron transport properties. The conductivity is directly related to the concentration of the conductive materials, while electron transport relies on tunneling effects. One-dimensional nanomaterials like nanowires (e.g., Ag NWs) possess a large aspect ratio, offering superior electrical conductivity compared to nanoparticles. Carbon-based materials such as activated carbon, carbon fiber, and graphene are also recognized for their ability to enhance hydrogel conductivity due to their reasonable conductivity, stability, and cost-effectiveness, forming 3D conductive networks within the polymer substrate through which electrons can pass via the conjugated structure. Despite their high specific surface area and mechanical excellence, these materials suffer from inherent hydrophobicity and poor solubility, leading to self-aggregation in aqueous media, posing a significant challenge for incorporation into hydrophilic hydrogel matrices [18,37]. This challenge can be mitigated to some extent by functionalizing the carbon surface, although this may potentially compromise the conductivity of the carbon frameworks [38,39].

In general, conductive gels are formed by incorporating various conductive components into different polymer substrates. Combining an insulating polymer with conductive fillers establishes structural integrity and moderate electrical conductivity. In situ polymerization with the infiltration of conductive monomers also yields effective conductive gels. Ionic conductive gels represent another approach to preparing conductive gels by dissolving ionic salts in polymers, a phenomenon ubiquitous in biological systems. Such types of conductive gels are suitable candidates for wearable or implanted bioelectronic devices [16,17,40].

**Figure 3:**
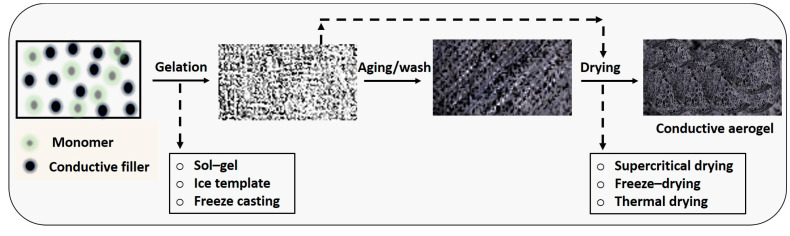
Conductive aerogels design principle.

Aerogels exhibit a porous structure containing micro-/meso-/macro-pores or nanofibrils, with gas serving as the dispersed phase. Rather than being classified based on materials or synthesis processes, the defining characteristic of aerogels lies in their structural composition. Essentially, an aerogel can be conceptualized as a framework permeated with air. Sometimes described as “frozen smoke”, “solid smoke”, “solid air”, or “blue smoke”, aerogels owe these names to their translucent appearance and the unique scattering of light within their matrix. The significance of aerogels extends to their electrocatalytic and electrical properties, which find application across various fields such as energy storage, energy conversion, supercapacitors, lightweight optics, and wearable devices. However, transitioning aerogel production to an industrial scale faces challenges, particularly in the drying process. The 3D structure of aerogels contributes significantly to their electrocatalytic activity, enhancing the conversion of different phases. This structure offers a larger surface area, increases the number of catalytic active sites, and improves mass and electron transport properties [41]. Aerogel formation involves the replacement of liquid with gas without collapsing the polymer networks. This process occurs in two distinct steps: gel formation and subsequent drying (Figure 3) [42]. The drying phase presents a significant challenge, as the surface tension of the solvent within the gel can promote the destruction of the gel structure during solvent evaporation.

Therefore, careful consideration of surface tension phenomena is essential during drying. Supercritical drying is a method that allows for the controlled removal of liquid. It involves subjecting the gel to high temperature and pressure, preventing the transition of liquid to gas from passing through any phase boundary, but rather surpassing the critical temperature and pressure within a closed container [43]. Carbon dioxide (with a critical temperature of 31.1 °C at 73.9 bar) and nitrous oxide are commonly employed for supercritical drying, where water is initially removed by washing and alcohol is replaced with liquified CO_2_. Subsequently, the CO_2_ is heated above its supercritical temperature, resulting in the production of dried gel materials [14,44]. Additionally, freeze-drying is utilized, which involves freezing the liquid and subsequently sublimating it. This strategy prevents the formation of a liquid–vapor interface, thereby avoiding structural damage caused by surface tensions. Thermal drying, another approach, involves using organic solvents to dry organic aerogels. Through this method, the wet gel’s pore solution is replaced with one or more low surface tension solvents, rendering the gels’ surfaces hydrophobic. This modification prevents shrinkage and structural deformation during drying, thereby safeguarding against damage [41,45,46].

Conductive polymers, unlike thermoplastics, have limited synthetic utility due to their inability to undergo thermoforming or molding processes. In contrast, conductive gels offer the advantage of hybridization with other materials. Moreover, they exhibit significant adhesion, swelling, and porosity, along with suitable or even superior mechanical properties compared to conductive polymers. Electronic conductive gels hold great promise for energy conversion and storage applications, such as batteries, supercapacitors, and fuel cells, owing to their robust mechanical strength, adhesion, and porosity. However, their stiffness imposes restrictions on their use in flexible or stretchable devices [47]. Additionally, exposure to air can lead to stability issues in electronic conductive gels due to dehydration. Meanwhile, ionic conductive gels find applications in energy generation, storage, flexible sensors, and electrochemical devices. Despite their utility, their mechanical strength is often insufficient, limiting their potential in various applications. Moreover, the use of potentially toxic solvents poses environmental challenges that need to be addressed [48,49].

## 3. Critical Factors of Gels

### 3.1. Mechanical Properties

The mechanical properties and durability of conductive gels are crucial factors in determining their applicability, particularly in terms of Young’s modulus, toughness, fracture strength, and strain resistance. These properties dictate the gel’s ability to withstand external forces and activities across various applications. Employing strategic approaches such as double networks, nano-composite network crosslinking, ion addition, slide ring structures, and the incorporation of hydrophobicity and topological networks play pivotal roles in preserving or enhancing the mechanical integrity of these gels [50,51]. The concept of a double network involves the intertwining of conductive polymers with other polymers. Within such gels, one network is tightly crosslinked, resulting in a rigid and brittle structure characterized by sacrificial bonds. Conversely, the second network is loosely crosslinked, endowing the gel with a soft and ductile nature. This dual-network architecture serves as a concealed reservoir of structural integrity, providing protection in the event of breakdown or fracture of the first network. Typically, the first network comprises conductive polymers, while the second network consists of flexible polymers such as PVA, PAM, and poly (acrylamide-co-hydroxyethyl methyl acrylate). The combination of conductive polymers and flexible polymers through in situ polymerization enhances the strength and toughness of the gel materials. Additionally, strategic modifications to synthesis strategies can further improve the mechanical properties of these materials [52,53]. For instance, the Li group utilized a PVA/PANI combination via vertical gradient freezing and cryopolymerization [54]. This approach leverages the crystallinity provided by PVA due to the unidirectional freezing process, thereby enhancing the mechanical strength of the gel materials. Consequently, these gels demonstrate superplastic performance, exhibiting a 100% recovery in tensile strength and a 50% recovery in compressive strength.

### 3.2. Rheological Properties

Rheology provides critical insights into the structure and behavior of gel systems, offering valuable information for their practical applications. Properties such as gelation time, storage modulus, loss modulus, and self-healing capabilities are key rheological parameters that determine the suitability of different gel materials for various applications [55,56]. Particularly in gel polymer electrolytes (GPEs), ionic conductivity is a crucial feature, and viscosity plays a significant role in this regard. Low-viscosity gels facilitate higher conductivity by creating efficient pathways within the polymer structure. The rheological properties of gels also influence their electrochemical performance, impacting flow and deformation behaviors. Within a limited range of deformation, gel materials exhibit both solid and liquid characteristics, demonstrating viscoelastic properties arising from the superposition of elastic and viscous flow. For GPEs used in electrochemical energy conversion or storage devices, it is essential for the gel to function as a perfect elastic network. Under low stress, GPE membranes behave like solids, transitioning to an elastic-viscous nature under increasing stress. Tackiness is another important consideration, as GPEs must adhere properly to electrode materials to optimize electrochemical performance [57,58]. The Huang group conducted a study on B crosslinked PVA/KOH/H_2_O GPE doped with GO, focusing on shear properties [59]. At ambient temperature, the GPE exhibited storage modulus (G′) and loss modulus (G″) values of 0.21 MPa and 0.022 MPa, respectively.

### 3.3. Printing Criteria of Gels

Three-dimensional printing of gels has emerged as a promising technique for efficiently patterning materials in three dimensions, particularly for fabricating flexible electronics intended for applications such as electronic skins and soft robotics. However, the successful implementation of 3D printing for ionotropic devices necessitates a delicate balance between various factors, including printability, ionic conductivity, shape fidelity, and stretchability [60].

The quality of 3D printing is contingent upon several key factors, namely printability, resolution, and shape fidelity. Printability, which refers to the smoothness of the printing process and the structural homogeneity of the printed object, can be enhanced through the utilization of light, temperature, and ion-responsive polymers. Notably, ink with high printability may exhibit diminished integrity post-printing. Viscous inks, such as methylcellulose–hyaluronan, chitosan–collagen, and chitosan blends, have demonstrated printing accuracies exceeding 95%. Light-based printing methods, while effective for photocuring under laser irradiation, may suffer from reduced resolution due to ink flow tendencies. Increasing the concentration of photoinitiator can enhance double bond conversion but may compromise structural integrity due to reduced cure depth [61,62].

Moreover, maintaining shape fidelity during the printing process is critical to preventing errors arising from gravity or external forces. Viscous inks play a crucial role in preserving shape fidelity, with rheological properties such as viscoelasticity and yield stress influencing the printing outcome. Optimization of printing parameters, including printing angle, oxygen concentrations, nozzle temperature, and the incorporation of nanoparticles such as silicate nanoplatelets, hydroxyapatite, and nanocellulose into the ink, is essential for achieving the desired shape fidelity [63,64].

Strategic approaches, such as photocrosslinking modification, click chemistry, and pre-, in situ, and post-crosslinking techniques, as well as the use of support baths and rheology tuning, along with moderate printing parameters, collectively contribute to improving the efficiency and quality of 3D printing processes for ionotropic devices [65].

## 4. Strategical Materials Design for Energy Applications

### 4.1. Electrochromic Devices

Electrochromic devices (ECDs) undergo reversible changes in optical properties upon the application of an external voltage, making them suitable for various potential applications, such as smart windows, anti-glare rearview mirrors, and displays [66]. Conventional ECDs typically comprise five layers, including two conductive substrates, an electrochromic layer, a counter layer, and an electrolyte layer. However, the rigid structure of these devices limits their applicability in flexible devices. Hydrogels with three-dimensional network polymers offer good flexibility and enable the development of stretchable electrochromic hydrogels. Nonetheless, conventional hydrogels often fail to withstand stress due to their lower mechanical properties arising from single polymeric networks [67,68,69,70]. To address this limitation and enhance mechanical properties, various strategies have been developed, including double-network interpenetrating hydrogels, nanocomposites, ion crosslinking, and topological hydrogels [71,72,73,74].

Polymer gel electrolytes (PGEs), characterized by their mechanically robust structure, are highly advantageous for integration into flexible and stretchable electronic devices. Unlike conventional liquid electrolytes, which are prone to leakage issues, polymer gel electrolytes offer enhanced reliability. Key considerations in the design of effective electrolytes for flexible electrochemical devices, including electrolyte-gated transistors, lithium-ion batteries, electrochromic devices, electroluminescence displays, and electrical skin, encompass not only ionic conductivity but also mechanical robustness. These factors collectively contribute to the optimal performance and longevity of such devices.

The practical application of intrinsically stretchable electrochromic materials encounters several challenges. These include the need for high-quality, full-color displays, issues related to coloration efficiency, concerns about toxicity and health effects, and the high production costs associated with synthesis, as well as the complexity of scaling up production. There is a growing demand for a universally applicable methodology that can address these challenges by enabling low-cost synthesis, rapid scaling up of the production process, and the attainment of multicolor tunable properties. Additionally, such a methodology should ensure desirable coloration efficiency, minimize energy consumption, and be environmentally and health-friendly [75].

Ion gels, when used as polymer electrolytes, exhibit the distinctive characteristics of block copolymers in combination with ionic liquids (ILs) at room temperature [76,77,78,79]. Typically, a conventional ion gel displays a mechanical elastic modulus in the order of a few kilopascals (kPa) and an ionic conductivity ranging between 1 and 10 mS cm^−1^ at room temperature [80,81]. These ion gels are composed of block copolymers that include both IL-compatible and IL-incompatible segments. ILs are favored for their non-volatility, high ionic conductivity, adjustable mechanical properties, and electrochemical stability. A common design principle involves the use of ABC triblock copolymers, wherein the A and C blocks are IL-insoluble terminal segments, and the B block is an IL-soluble middle segment. In various blended triblock copolymers and IL systems, the IL-insoluble components tend to aggregate into spheres to minimize surface area, thereby enhancing the mechanical properties of the gels. Concurrently, the IL-soluble middle blocks, when swollen with ILs, provide efficient pathways for electrochemical reactions. Crucially, for optimal activity, the middle block must directly connect with the IL-insoluble spheres, maintaining a concentration of 10–20 wt% of the block copolymers [82,83]. This necessitates a careful design of polymer gel electrolytes (PEGs) to improve the mechanical modulus without compromising ionic conductivity. Achieving this goal may involve selective chemical crosslinking of the IL-insoluble blocks, though synthesizing such a system presents significant challenges.

**Figure 4:**
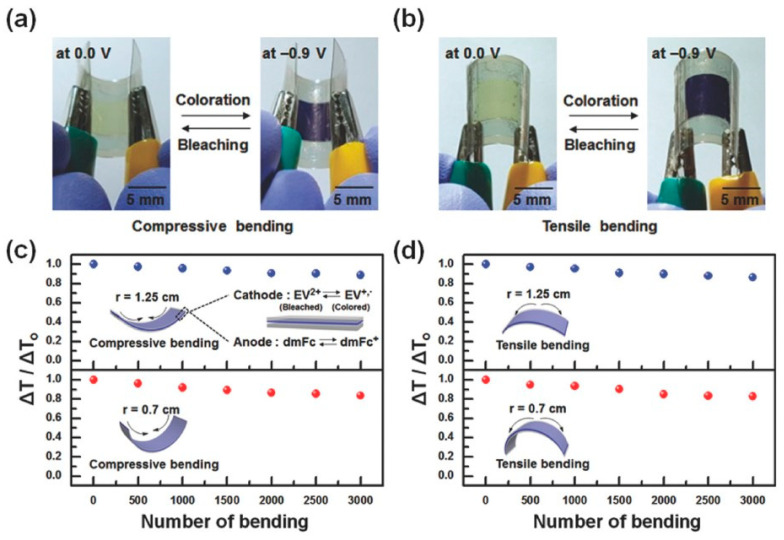
Images depicting electrochromic devices (ECDs) in bleached and colored states subjected to (**a**) compressive and (**b**) tensile bending, with a bending radius of approximately 0.7 cm; changes in ΔT/ΔT_o_ over bending cycles are shown for (**c**) compressive and (**d**) tensile bending, utilizing two distinct bending radii of 1.25 and 0.7 cm (adapted with permission from Ref. [84], Copyright, 2018 John Wiley and Sons).

Moon’s groups have designed and synthesized a mechanically robust conductive random copolymer, poly[styrene-ran-1-(4-vinylbenzyl)-3-methylimidazolium hexafluorophosphate] (P[S-r-VBMI][PF6]), with a mole fraction of PS ≈ 0.80, using reversible addition–fragmentation chain transfer polymerization (Figure 4) [84]. A homogenous ion gel was then prepared by blending 40 wt% of P[S-r-VBMI][PF6] with 60 wt% of [EMI][TFSI], providing a mechanical elastic modulus of 0.105 MPa and an ionic conductivity of 1.15 mS cm^−1^, suitable for application in electrochromic devices. The mechanical strength of the P[S-r-VBMI][PF6]–gel (0.105 MPa) was found to be considerably higher than that of the homopolymer-based gel P[VBMI][PF6] (0.0208 MPa) (Table 1). Despite the improvement in mechanical properties, the modified gel exhibited lower ionic conductivity (1.15 mS cm^−1^) [84]. The homogeneous ion gel, consisting of a blend ratio of 40 wt% P[S-r-VBMI][PF6] and 60 wt% [EMI][TFSI], plays a crucial role in forming a uniformly physically crosslinked gel, which exhibits mechanical resilience. In this system, poly[styrene-ran-(4-vinylbenzyl chloride)] (P(S-r-VBC)), composed of PS and PVBC, is insoluble in [EMI][TFSI]. Through precise synthesis engineering, PVBC is functionalized with imidazolium and subjected to ion exchange with chloride to hexafluorophosphate, rendering it soluble in [EMI][TFSI]. This maintains the ionic conductivity, while the insoluble mole fraction (0.78) of PS contributes to the enhanced mechanical resilience.

The Zhang group has developed a promising prototype device by integrating an intrinsically stretchable electrochromic hydrogel (polyacrylamide, PAAM) into the electrode setup [85]. This design features the utilization of Au nanosheets as the counter electrode and Ag nanowires as the cathode layer. The device demonstrates impressive performance metrics, including a coloration efficiency of 291.35 cm^2^ C^−1^ for hydrogel–R and 152.18 cm^2^ C^−1^ for hydrogel–G, as well as excellent reversibility (>200 cycles) and a short open time (<100 ms) when utilizing ITO as the electrode glass. Moreover, the device exhibits a controllable color change from yellow to red to brown, easily manipulated reversibly under varying tensile strengths [85]. The design features of the electrochromic hydrogel for wearable technology showcase promising potential applications, as evidenced by several key attributes. Firstly, the inclusion of Ag nanowires in the cathode serves to prevent oxidation, ensuring efficient conductivity and high transparency. This combination facilitates the high-quality display of information. Additionally, the use of Au nanosheets in the counter electrode contributes to good conductivity and electrochemical stability, resulting in eye-friendly color reproduction of high quality. The PAAM hydrogel component demonstrates impressive characteristics, including over 95% transmittance and high ionic conductivity, even when subjected to stretching processes that only marginally decrease conductivity. Furthermore, the incorporation of p-BQ (P-benzoquinone) as the electroactive base, alongside responsive molecules such as M-R (red sodium salt) or thymol blue sodium salt (M-G), is well-dispersed within the PAAM hydrogel. This dispersion enhances the efficiency and performance of the electrochromic system.

**Figure 5:**
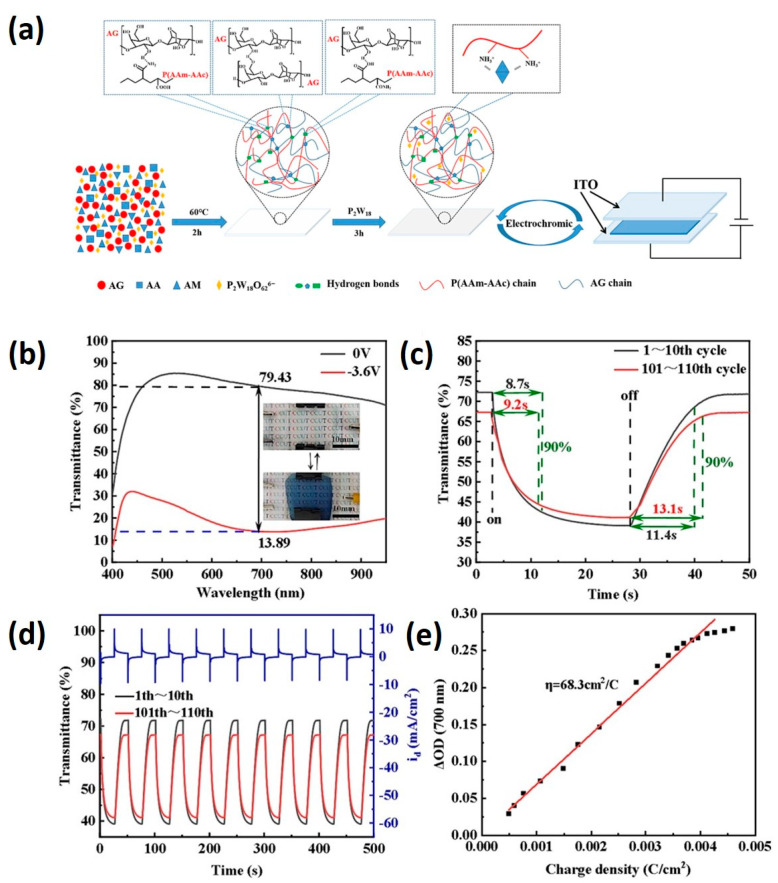
(**a**) Preparation process of AG/P(AAm–AAc)/P_2_W_18_ double-network electrochromic hydrogel. Electrochromic properties of AG/P(AAm–AAc)-10/P_2_W_18_ hydrogel electrochromic device, (**b**) optical transmittance of the tinted/faded state of the hydrogel electrochromic device, (**c**) tinting time and fading time of the hydrogel electrochromic device, (**d**) cycling stability of the hydrogel electrochromic device, and (**e**) coloration efficiency of the hydrogel electrochromic device (adapted with permission from Ref. [86], Copyright, 2024 Elsevier Ltd.).

The Song group employed double-network hydrogels, which combine two interpenetrating networks utilizing agar and poly (acrylamide–acrylic acid) copolymer (Figure 5) [86]. The first network consists of physically crosslinked agar, while the second network comprises chemically crosslinked poly (acrylamide–acrylic acid) (PAAm/PAAc). Mechanical properties are finely tuned by adjusting the copolymerization ratio of (acrylamide–acrylic acid). When incorporating P_2_W_18_ into the hydrogel, designated as 10% (AG/P(AAm-AAc)-10/P2W18), the resulting hydrogel exhibits maximum elongation at break and fracture stress. Upon the application of an external voltage, the colorless hydrogel transitions to blue, with an optical contrast of up to 65.54% at 700 nm [86].

### 4.2. Water Splitting

Metal–organic gels (MOGs) have been utilized for the oxygen evolution reaction (OER) in hydrogen generation. These MOGs benefit from synthesis under mild reaction conditions, including aqueous-phase processes, normal temperature and pressure, and short reaction times, to form coordination-driven supramolecular polymers. Their 3D hierarchical structures exhibit low density and diverse functional sites centered around metals [87,88]. Mechanistically, metals and ligands form gel precursors through self-assembly, which subsequently organizes into fibrous, flake-like, or granular structures, generating a 3D network through intermolecular hydrogen bonding, van der Waals forces, or π–π stacking interactions [89,90].

Zhang et al. developed a Ni0.6Fe0.4–MOG specifically for enhancing OER catalytic activity, demonstrating an overpotential of 289 mV at a current density of 10 mA cm^−^^2^ [91]. Additionally, the kinetics of OER, indicated by the Tafel slope, revealed a significantly lower value for the synthesized MOGs (33 mV dec^−1^) compared to RuO_2_ (68 mV dec^−1^), highlighting the efficiency and potential of MOGs in OER applications [91].

**Figure 6:**
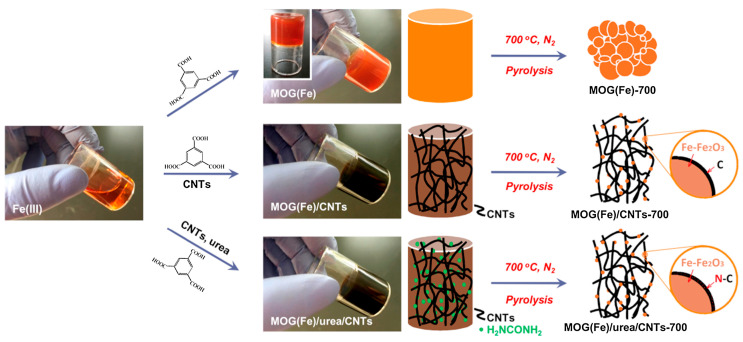
Preparation of Fe–Fe_2_O_3_ nanoparticles embedded within an N-doped carbon matrix from the MOG(Fe), MOG(Fe)/CNTs, and MOG(Fe)/urea/CNTs (adapted with permission from Ref. [93], Copyright 2017 Elsevier Ltd.).

The Li group has reported on the development of P-doped Ni-Mo bimetal aerogel as a bifunctional water-splitting electrocatalyst [92]. Bimetallic electrocatalysts have demonstrated promising catalytic performance attributed to electronic modulation, which regulates catalytic activity through intermediate species and controls reaction kinetics. Phosphorus (P) acts as an active adsorbent for protons and oxygen-containing intermediates, leveraging its higher electronegativity to attract electrons from the metal surface. This facilitates the reduction in energy barriers for hydrogen and oxygen desorption during the catalytic processes of HER (hydrogen evolution reaction) and OER (oxygen evolution reaction). The incorporation of P doping modulates the in situ electronic structure without altering intrinsic properties. The bifunctional Ni-Mo-P aerogel catalyst exhibits an HER performance of 69 mV at 10 mA cm^−2^ and an OER performance of 235 mV at 10 mA cm^−2^, resulting in excellent water splitting efficiency at a low cell voltage of 1.46 V at a current density of 10 mA cm^−2^. The porous aerogel structure provides abundant exposed active sites, facilitating efficient mass transport within interconnected channels. The hydrophilicity of Mo sites enhances the desorption of H_2_ at the gas–liquid–solid interface. P doping further modulates electron densities within the Ni-Mo metallic alloy, increasing the number of empty d-orbitals and promoting the adsorption of OH^−^ and H^+^ ions. Both Mo-alloying and P-doping modify the D-band density of Ni, optimizing *H bond strength and reducing energy barriers for the adsorption of intermediate species, ultimately leading to reduced energy barriers for water splitting [92]. In the study conducted by the He group; an innovative approach was employed utilizing urea and carbon nanotubes to decorate MOGs with iron (Fe) as a novel sacrificial precursor (Figure 6) [93,94]. This method facilitated the synthesis of Fe–Fe_2_O_3_ nanoparticles embedded within an N-doped carbon matrix, aimed at enhancing the oxygen reduction reaction (ORR) catalysis. The resultant catalyst demonstrated impressive catalytic performance, characterized by an onset potential of 0.92 V and a half-wave potential of 0.72 V. Furthermore, it exhibited notable durability, maintaining 91.7% of its initial current after continuous operation for 20,000 s in ORR conditions. This investigation underscores the potential of MOGs as a versatile and effective precursor for the fabrication of catalysts with highly dispersed and homogeneously distributed active sites, optimizing the structural and compositional qualities essential for advanced ORR catalysis [93].

Transition-metal-based catalysts are extensively recognized as promising electrocatalysts for water splitting owing to their abundance and cost-effectiveness. These catalysts are available in various forms, including nanoparticles, oxides, hydroxides, carbides, phosphides, and nitrides [95,96]. However, their efficiency is often hampered by issues such as limited exposure of active sites, insufficient electrolyte contact, and agglomeration. Incorporating heteroatoms into a carbon matrix containing transition metals emerges as a strategic design to enhance catalytic performance while ensuring durability under stringent electrocatalytic conditions. This combination of transition metal active species with a conducting carbon matrix not only improves conductivity and corrosion resistance but also, through heteroatom doping, creates an uneven charge distribution on the doped carbon matrix [97,98,99]. This results in a higher electron density near the Fermi level of the transition metal, thereby emulating the electrocatalytic performance of noble metals due to the altered electronic band structure [41,100].

**Figure 7:**
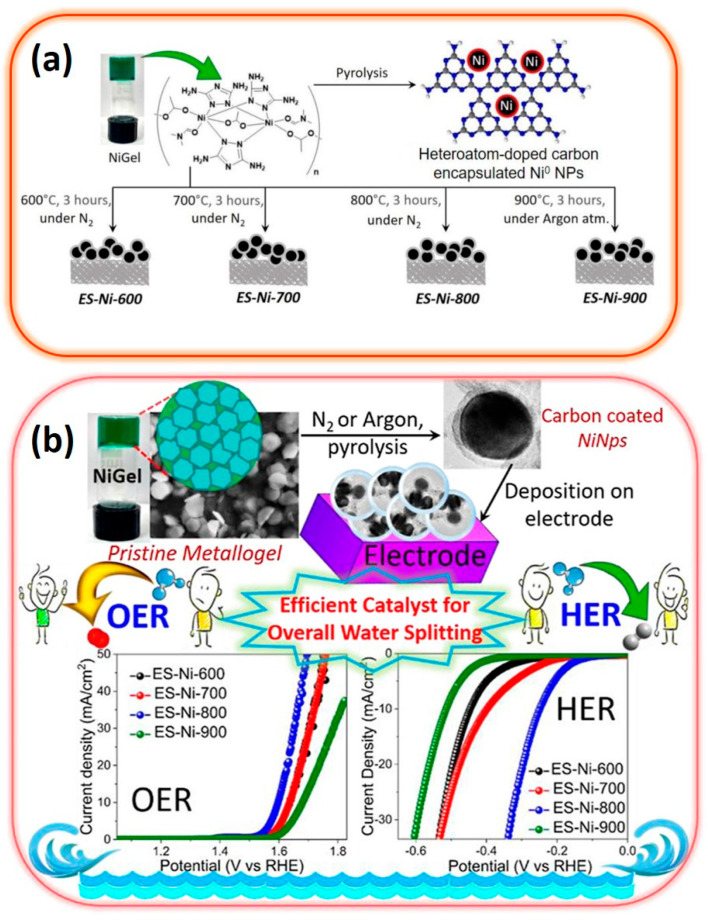
(**a**) Illustration outlining the synthesis process of Ni (0)–nanoclusters encapsulated in heteroatom–doped carbon onions derived from Ni–gels, and (**b**) utilization of the resulting material as a bifunctional electrocatalyst for water splitting (adapted with permission from Ref. [104], Copyright 2023 Elsevier Ltd.).

Currently, the in situ confinement of transition metal nanoparticles through synthetic strategies often relies on laser deposition, rapid thermal annealing, and plasma treatment [101]. These methods necessitate specialized, complex, and costly equipment. Moreover, the doping of carbon with heteroatoms requires the presence of a pre-assembled organic template that allows for composition tuning [102,103]. In this context, Saha et al. have explored the use of a supramolecular Ni (II)–triazole gel as a novel synthetic precursor for creating a bifunctional electrocatalyst targeting both OER and HER (Figure 7) [104]. This innovative approach leads to the formation of Ni (0) nanoclusters enveloped within heteroatom-doped carbon onions derived from the Ni (II)–triazole gel. This configuration demonstrates significant catalytic efficiency, requiring low overpotentials of 360 mV for OER and 250 mV for HER, along with Tafel slopes of 69 mV dec^−1^ and 115 mV dec^−1^, respectively [104].

**Figure 8:**
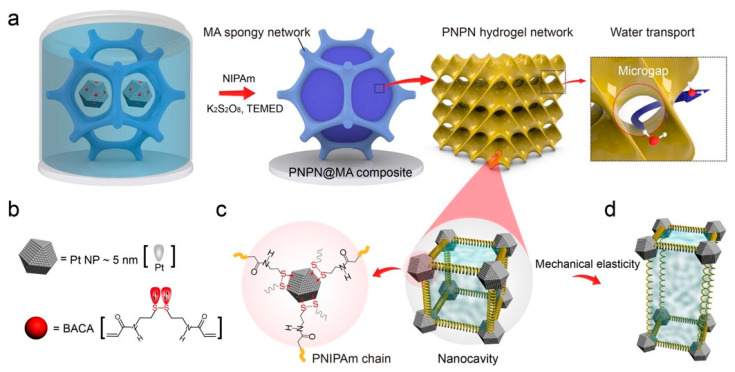
(**a**) Illustration depicting the fabrication process of PNPN@MA composite with a double-confinement-cavity structure via in situ polymerization, (**b**) schematic representation of the BACA molecule functioning as both a donor ligand and a metalloid acceptor for Pt NPs, forming Pt-SR coordination bonds, (**c**) schematic depiction of the dynamic catalyst surface within the nanocavity, and (**d**) diagram illustrating the mechanism underlying the intrinsic elasticity of the nanocavity skeleton during the stretch/release process (adapted with permission from Ref. [107], Copyright 2022 John Wiley and Sons).

Catalysts may lose their activity due to surface poisoning, but they can be regenerated to their original levels through deactivation. This reversible interaction produces stable and reproducible active sites within a confined architecture [105,106]. Qin et al. have innovatively demonstrated the synthesis of a regenerative hydrogel photocatalyst for water splitting, incorporating metal–thiolate coordination to induce nanocavities within a robust, micro-sized spongy network (Figure 8) [107]. This study advances double-confinement cavity engineering to create an intelligent hydrogel. In situ polymerization of Pt–thiolate coordination forms a polymeric gel network that transitions into a robust spongy framework. The hydrogel benefits from size confinement effects within the gel network and synergistic spatial confinement of the spongy network, exhibiting significant H_2_ production of 3.24 mmol h^−1^ g^−1^ under visible light irradiation. The in situ growth of conductive polymers on the hydrogel enhances photogenerated charge separation efficiency. The optically dynamic Pt–S coordination is utilized to regenerate the deactivated hydrogel, where the sulfur-terminated polymer chains act as dynamic brushes to remove carbonaceous deposits from the metal catalyst surface and regulate the nanocavities under NIR irradiation. Consequently, H_2_ evolution increases threefold compared to the pristine activity, from 26% to 72% [107].

Conductive polymer gels exhibiting high conductivity and favorable hydrophilicity serve as promising substrates for electrochemical reactions, particularly those involving gas, liquid, and solid phases in the oxygen evolution reaction (OER) process. From a fundamental perspective, the efficient contact between electrolyte and electrocatalyst is pivotal in facilitating electrolyte transport on the catalyst surface [108,109]. This can be achieved by designing super-hydrophilic catalysts. Hu et al. employed phytic acid (PA)-doped polypyrrole (PPy) coated onto carbon cloth via dip coating [110]. Phytic acid contains certain amounts of positive charge, which can activate the OER process due to its appropriate binding energy with intermediates. PPy serves as an efficient structure that facilitates mass and charge transfer during the OER process. Notably, the PA-PPy/CC conductive hydrogel demonstrates significant activity towards OER, exhibiting a small overpotential of 340 mV at 10 mA cm^−2^, a Tafel slope of 54.9 mV dec^−1^, and a notable stability of 20 h [110].

### 4.3. Batteries

Rechargeable Zn-ion batteries are considered promising prospects for the post-lithium battery era due to their high theoretical capacity (~820 mAh g^−1^), low redox potential (–0.76 vs. SHE), abundant zinc reserves, non-toxicity, and affordability [111]. However, in operational conditions, the strong interaction between Zn^2+^ ions and water molecules in the surrounding solvent significantly hinders the mobility of ion carriers in the electrolyte, resulting in slow kinetics of the process [112]. To address this issue, the utilization of water-in-salt and hydrate melt electrolytes has been proposed, which partially mitigates the problem but does not eliminate it entirely. Additionally, it is essential to consider the complex and costly preparation process associated with these electrolytes [113,114].

Polymer gel electrolytes (PGE) offer an alternative approach characterized by non-flowing behavior and stability for Zn-ion batteries (ZIBs). The Tan group has designed a hydrogel electrolyte based on covalent organic frameworks (COFs), termed TCOF-S-gel, which precisely integrates PGE and a single-ion conductor [115]. The incorporation of a single-ion conductor effectively eliminates electrode polarization, reduces the proportion of anion migration, and hinders side reactions in the batteries. However, the challenge lies in the insufficient ion conductivity attributed to the strong interaction between cations and cation receptors.

To address this, COFs modified with sulfonic acid groups have been introduced to reduce the electrostatic interaction between Zn^2+^ ions and the sulfonic acid groups [115]. This modification, facilitated by the solvation effects of polyacrylamide, promotes the decoupling of Zn^2+^ cations and their formation into a Zn^2+^ stair with the sulfonic acid groups on the COF pore wall. The resulting TCOF-S-gel exhibits enhanced mechanical properties, and the strong hydrogen bonding of the sulfonic acid groups within the COF pore channels with water molecules aids in water retention. The TCOF-S-gel exhibits an ion conductivity of 27.2 mS cm^−1^ and a Zn^2+^ transference number of 0.89. The assembled full battery, consisting of a Zn||TCOF-S-Gel||MnO_2_ configuration, demonstrates a discharge capacity of 248 mAh g^−1^ at a 1C. It maintains stability with almost unchanged performance after 1,400 cycles, exhibiting low polarization voltage (244 mV) and a minimal difference in redox potential difference (305 mV) at the 1C [115].

Zinc–air batteries (ZABs), hailed as flexible devices, encounter severe performance degradation under low-temperature conditions due to the electrocatalysis principle in their aqueous environment. To address this challenge, the Chen group devised a novel 1D fiber, bamboo-structured electrocatalyst and a freezing-tolerant hydrogel electrolyte. This innovative design showcases remarkable performance, yielding a capacity of 691 mAh g^−1^ and an energy density of 798 Wh kg^−1^ at –20 °C [116]. Impressively, the device exhibits substantial capacity and energy density retention of 92.7% and 87.2%, respectively, when transitioning from 25 °C to –20 °C. The distinctive viscosities and surface tensions of polyacrylonitrile (PAN, outer layer) and polyvinylpyrrolidone (PVP, inner layer) species segregate into two layers along the radial directions of the fiber without intermixing, resulting in well-defined 1D fibrous structures. During pyrolysis at 900 °C, the inner PVP decomposes and migrates towards the outer PAN layer, creating abundant porosity in the fiber shell conducive to enhanced catalytic activity at the interior conductive fibers. Moreover, the highly conductive hydrogel, featuring polarized terminal groups, imparts anti-freezing properties, ensuring adaptability to cold temperatures [116].

Polymer hydrogels are compromised by parasitic hydrogen evolution reactions (HERs) due to the narrow electrochemical stability window of water (1.23 V) and the dissolution of metal cations from the cathode. A strategy in liquid electrolyte engineering that minimizes solvent content while maximizing salt concentration can lead to the formation of salt-derived solid electrolyte interphases [117,118]. Ideally, polymer hydrogel electrolytes with a high salt concentration should facilitate salt dissolution and provide a mechanically stable, crosslinked network. However, protic polymer matrices such as polyamides, polyalcohols, and polyacrylic acids contain extensive intra- and intermolecular hydrogen bonding networks. As a result, the polymer component of hydrogel electrolytes may not effectively contribute to salt dissolution, leading to stable salt concentrations and phase separation between the polymer and salt [119,120,121].

**Figure 9:**
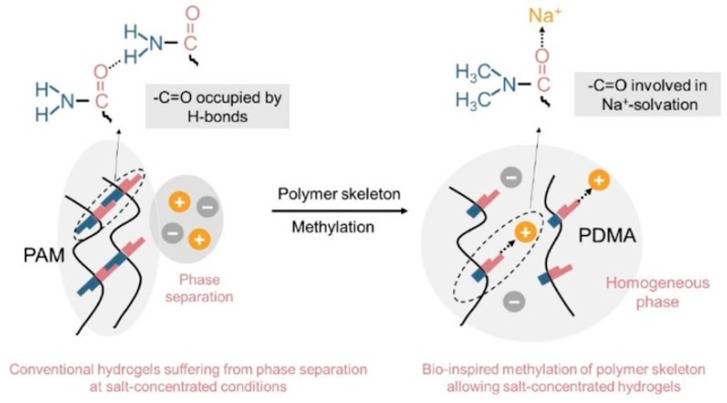
Schematic illustration of the polymer structure design to promote cation solvation for the salt-concentration hydrogel electrolytes (adapted with permission from Ref. [122], Copyright 2013 John Wiley and Sons).

To address the challenge of phase separation, the Cui group implemented a methylation modification strategy on polymer matrices to maintain concentrated hydrogel electrolytes (Figure 9) [122]. By modifying polyacrylamide (PAM) with sodium salt and a calculated amount of water through methylation, protic PAM was transformed into aprotic poly (N, N-dimethylacrylamide) (PDMA). This modification involves replacing the hydrogen-bond donor (–NH_2_) in PAM with –N(CH_3_)_2_, which reduces or weakens intra- and intermolecular hydrogen bonds and enhances the solvation of Na^+^ ions at the unoccupied –C=O sites of the polymer structure. Consequently, this approach yields a stable hydrogel electrolyte with a high salt content of 44 mol%. In this optimized system, the ratio of salt to water is 1:1, ensuring that all water molecules and polymer units are confined within the primary solvation shell of the cations, thereby enhancing the electrolyte’s stability and electrochemical performance [122].

**Figure 10:**
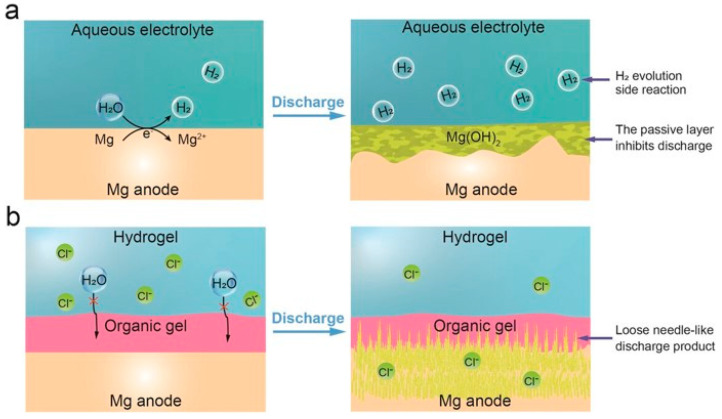
Discharge process of Mg–air batteries in various electrolytes. (**a**) Conventional aqueous electrolytes result in H_2_ evolution at the anode–electrolyte interface, leading to the formation of a dense Mg(OH)_2_ passive layer and eventual battery failure, and (**b**) the dual–layer gel electrolyte proposed here effectively prevents Mg anode corrosion and promotes the formation of loose needle–like discharge products, sustaining the discharge process until complete consumption of the Mg anode (adapted with permission from Ref. [126], Copyright 2024 John Wiley and Sons).

The Mg air battery is considered one of the most promising flexible energy storage devices, as it poses no harm to the human body, making it an alternative candidate for use on the skin and inside the body [123]. However, the corrosion of Mg batteries and the low utilization of the Mg anode are critical issues hindering the progress of these devices. In general, in an aqueous electrolyte, Mg corrodes to Mg (OH)_2_ and produces H_2_. Here, electrons pass directly through the H^+^ ions instead of the external circuit, resulting in an efficiency problem characterized by capacity loss. Additionally, the formation of an insoluble dense passive layer on the Mg anode surface hinders the connection between the Mg anode and the electrolyte, consequently reducing the efficiency of Mg utilization [124,125]. The Zhang group has addressed these two issues simultaneously by using a dual-layer gel electrolyte (poly (ethylene oxide) (PEO) organic gel and crosslinked polyacrylamide (PAM) hydrogel) [126]. This electrolyte prevents the corrosion that leads to the formation of a passive layer on the Mg anode, while also allowing loose needle-like discharge products to be maintained during the discharge process (Figure 10). As a result, the Mg utilization rate reached 99.3%, with a specific capacity and energy density of 2190 mAh g^−1^ and 2282 Wh kg^−1^, respectively [126].

**Figure 11:**
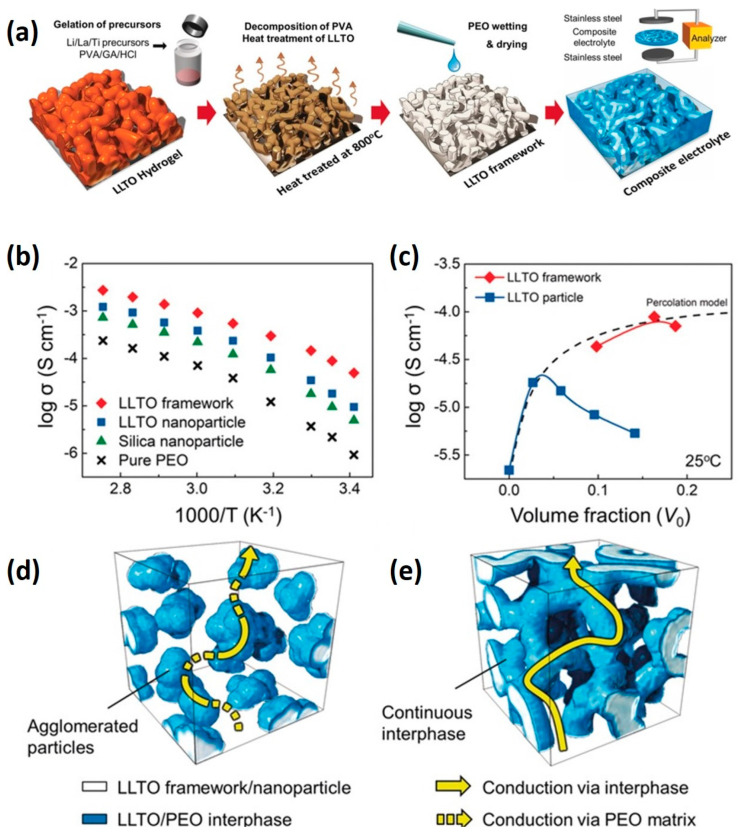
(**a**) Schematic outlining the synthesis process of LLTO framework composite electrolytes, including LLTO hydrogel synthesis, decomposition and heat treatment, PEO wetting and drying, and electrochemical analysis of the composite electrolyte; (**b**) comparison of ionic conductivity among LLTO framework, LLTO nanoparticle, and silica particle composite electrolytes; (**c**) representation of the percolation model (black dashed line) alongside conductivity data of composite electrolytes featuring LLTO nanoparticles (blue) and framework (red); schematic illustration depicting potential conduction mechanisms in composite electrolytes with (**d**) agglomerated nanoparticles and (**e**) continuous 3D framework structures (adapted with permission from Ref. [133], Copyright 2018 John Wiley and Sons).

In the realm of lithium-ion battery applications, solid-state electrolytes are increasingly utilized to supplant liquid electrolytes, mitigating flammability risks and curtailing the formation and growth of lithium dendrites. These solid polymer electrolytes, typically comprised of high molecular weight polymers such as polyethylene oxide (PEO) or polyacrylonitrile (PAN) combined with lithium salts, offer flexibility and cost-effectiveness in processing. However, polymer crystallization at room temperature often leads to inadequate lithium-ion conductivity (ranging from 10^−6^ to 10^−8^ S cm^−1^ at 20 °C) [127,128]. Incorporating non-lithium-ion conductive fillers like SiO_2_, TiO_2_, or Al_2_O_3_ induces an amorphous phase within the polymer, thereby enhancing lithium-ion conductivity. Furthermore, alternative fillers such as Li_7_La_3_Zr_2_O_12_ (LLZO) or Li_10_GeP_2_S_12_ (LGPS) can augment conductivity owing to increased interphase volume [129,130]. Nonetheless, an optimal filler ratio is pivotal as excessive filler content can lead to agglomeration and subsequent reduction in conductivity. Nanostructured fillers have emerged as a solution to lower percolation thresholds through their high aspect ratios, thereby enhancing lithium-ion conductivity, although they can also promote agglomeration, posing a challenge. Generally, a 3D percolated network with high filler concentrations facilitates rapid conduction along the interphase, while maintaining a higher filler-to-polymer ratio ensures improved electrochemical stability and device safety [131,132]. The Yu group, for instance, has leveraged 3D nanostructured hydrogel hierarchical frameworks to facilitate ion/electron transport [133]. Through the design of pre-percolated Li_0.35_La_0.55_TiO_3_ (LLTO) continuous frameworks via gelation of LLTO and hydrogel (polyvinyl alcohol/glutaraldehyde; PVA/GA) followed by heat treatment, they achieved a 3D percolated structure that bolstered lithium-ion conductivity to 8.8 × 10^−5^ S cm^−1^ at room temperature, concurrently enhancing thermal and electrical stability (Figure 11). Here, the LLTO frameworks function as interconnected 3D nano-fillers, forming a continuous interphase that impedes filler agglomeration, thereby facilitating lithium-ion conduction while ensuring thermal and electrical stability [133].

### 4.4. Anti-Freezing Conductive Gels

**Figure 12:**
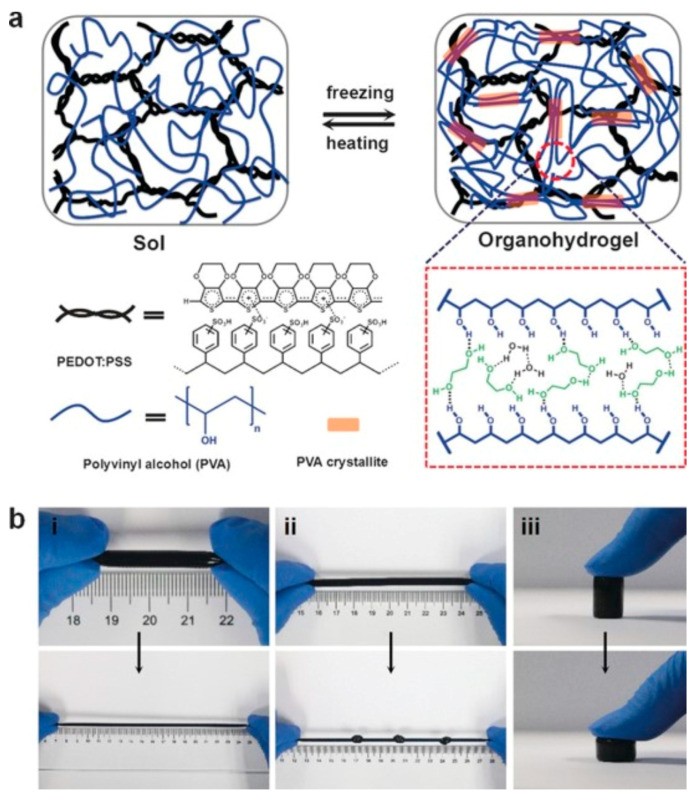
(**a**) Schematic representation of the preparation process and structural characterization of the anti-freezing conductive organohydrogel; (**b**) photographic demonstration of the anti-freezing conductive organohydrogels showcasing their resilience to (i) extensive stretching, (ii) knotted stretching, and (iii) compression (adapted with permission from Ref. [135], Copyright 2017 John Wiley and Sons).

The conventional conductive hydrogels, which rely on pure water at subzero temperatures, suffer from a loss of conductivity, thereby restricting their utility in low-temperature settings [134]. To overcome this limitation, freezing-tolerant high-strength conductive hydrogels were developed employing an anti-freezing binary solvent system. Within this framework, the Liu group synthesized conductive organohydrogels incorporating conductive polymers such as PEDOT:PSS in a water/ethylene glycol solvent (EG) [135]. Remarkably, these conductive organohydrogels demonstrate stable flexibility and strain sensitivity even at –40 °C. The solvent molecules, specifically ethylene glycol, establish hydrogen bonds with the PVA chain, thereby facilitating the crystallization of PVA and consequently enhancing the mechanical strength of the organohydrogels (Figure 12). Furthermore, the presence of non-covalent crosslinking networks endows the conductive organohydrogels with self-healing and remodeling capabilities [135].

In the realm of battery technology, zinc-ion batteries present promising avenues owing to their inherent safety features, eco-friendliness, and cost-effectiveness in fabrication. However, a critical requirement for these systems lies in their ability to maintain performance across a broad spectrum of temperatures. To address this challenge, hydrogel electrolytes emerge as a viable solution, bolstered by the incorporation of anti-freezing additives such as calcium chloride (CaCl_2_) and lithium chloride (LiCl), among others. While these additives effectively lower the freezing point, they often entail a trade-off by compromising mechanical properties such as tensile strength, fracture toughness, and elongation at break. This compromise stems from the salting-in effect, which induces polymer aggregation and subsequently deteriorates mechanical integrity [136,137,138].

Recognizing these challenges, the He group endeavors to engineer poly (vinyl alcohol) (PVA) hydrogel electrolytes tailored specifically for zinc-ion batteries [138]. These hydrogels exhibit a unique open-cell porous structure characterized by strongly aggregated polymer chains and disrupted hydrogen bonds among free water molecules. Remarkably, their hydrogel demonstrates an impressive tensile strength of 15.6 MPa and robust weather tolerance down to temperatures below −77 °C.

The strategy capitalizes on the synergistic interplay between co-nonsolvency and salting-out effects during hydrogel fabrication. The salting-out effect facilitates chain aggregation while maintaining a high water content, thereby enhancing the toughness of the hydrogels through the promotion of chain aggregations by potassium ions (K^+^) and acetate ions. Concurrently, co-nonsolvency promotes chain aggregation and facilitates the formation of a porous structure within the polymer network, vastly improving mass transport properties and reducing overpotential by an order of magnitude. Key to their approach is the utilization of potassium acetate (KAc) and zinc acetate (ZnAc_2_) as salting-out agents, conferring anti-freezing properties crucial for low-temperature operation. The anti-freezing ability is augmented by disrupting hydrogen bonds, with higher salt concentrations favoring both anti-freezing capability and mechanical strength, while lower concentrations optimize battery performance [138].

Furthermore, the He group demonstrates that higher concentrations of PVA solution (at a 6:4 ratio) and higher molecular weights yield stronger hydrogels, thus enhancing overall performance. Through the strategic integration of salting-out and co-nonsolvency effects with anti-freezing salts, their hydrogel electrolytes offer unparalleled low-temperature tolerance, substantial mechanical strength, and enhanced mass transport properties, underscoring their potential for next-generation zinc-ion battery applications [138].

### 4.5. Supercapacitors

Flexible supercapacitor electrodes, including carbon, metal oxide, conductive polymer, and composite variants, are crucial for sustaining mechanical strain when coated onto elastic yet electrochemically inactive substrates, such as PDMS, cotton sheets, or rubber fibers [139,140,141,142,143,144,145]. However, these substrates inherently bear a significant weight and volume, rendering them unsuitable for flexible devices [146]. To address this challenge, conductive polymer-based hydrogels offer a promising alternative capable of withstanding mechanical deformation. While materials like PEDOT–PSS, polypyrrole, and polyaniline have been considered for flexible supercapacitors, their tensile strength typically falls below 1 MPa [147,148].

Recognizing this limitation, the Ma group hypothesized that a combination of rigid conductive polymers with a soft hydrophilic polymer could yield a strong and robust conductive polymer hydrogel, partially fulfilling the desired criteria [149]. Their design involved employing PVA as the soft polymer and PANI as the rigid polymer, with boric acid facilitating gelation and ensuring gel robustness. Boronic acid was chosen as the functional group for crosslinking PVA and PANI, resulting in the production of the conductive polymer hydrogel termed PPH. PPH exhibited a mere improvement in tensile strength, reaching 5.3 MPa, but the desirable range of 30–50 MPa for practical applications in flexible devices [150,151]. The polymer utilized in flexible solid-state capacitors demonstrated an electrochemical capacitance of 306 mF cm^−2^ (equivalent to 153 F g^−1^) and an energy density of 13.6 Wh kg^−1^, while maintaining 100% capacitance retention after 1000 mechanical folding cycles and 90% capacitance retention during galvanostatic charge–discharge cycles [149].

### 4.6. Self-Healing Gels

The utilization of conductive gels in multifaceted applications such as biomedicine, bioelectronics, electronic skins, and similar fields, characterized by their electrical conductivity, softness, and flexible mechanical properties, is essential. However, conventional gels often lack the self-healing capability required for such applications to restore their original state after being damaged, thereby compromising reliability and longevity.

**Figure 13:**
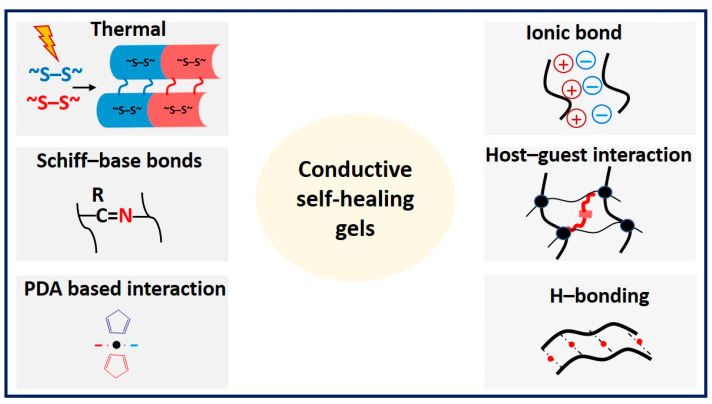
Illustration depicting the self-healing mechanism commonly found in conductive hydrogels.

Conductive gels exhibit self-healing properties in response to external stimuli such as heat, pH changes, the presence of self-healing agents, and autonomous interactions of materials facilitated by dynamic chemical bonds, noncovalent interactions, metal coordination interactions, hydrogen bonding interactions, interactions between polymer nanomaterials, and host–guest interactions [152] (Figure 13).

Mechanical force sensing and self-healing are prerequisites in robotics applications and optical force measurement. Achieving all these properties in a single material presents significant challenges. The Xu group has fabricated fluorescent-responsive self-healing hydrogels (composed of PVA/chitosan/agarose/tetraborate/glycerol/quantum dots of carbon, MXene) with a triple network structure [153]. These hydrogels exhibit impressive characteristics, including 100% recovery of tensile strength after 30 s of healing in air and 90% recovery after 60 s of healing in water. Additionally, the materials can withstand 1800° rotation without breaking at the healed site. This advancement in material performance addresses the limitations of conventional hydrogels, such as long healing times, poor healing performance, and unstable optical properties [153].

**Figure 14:**
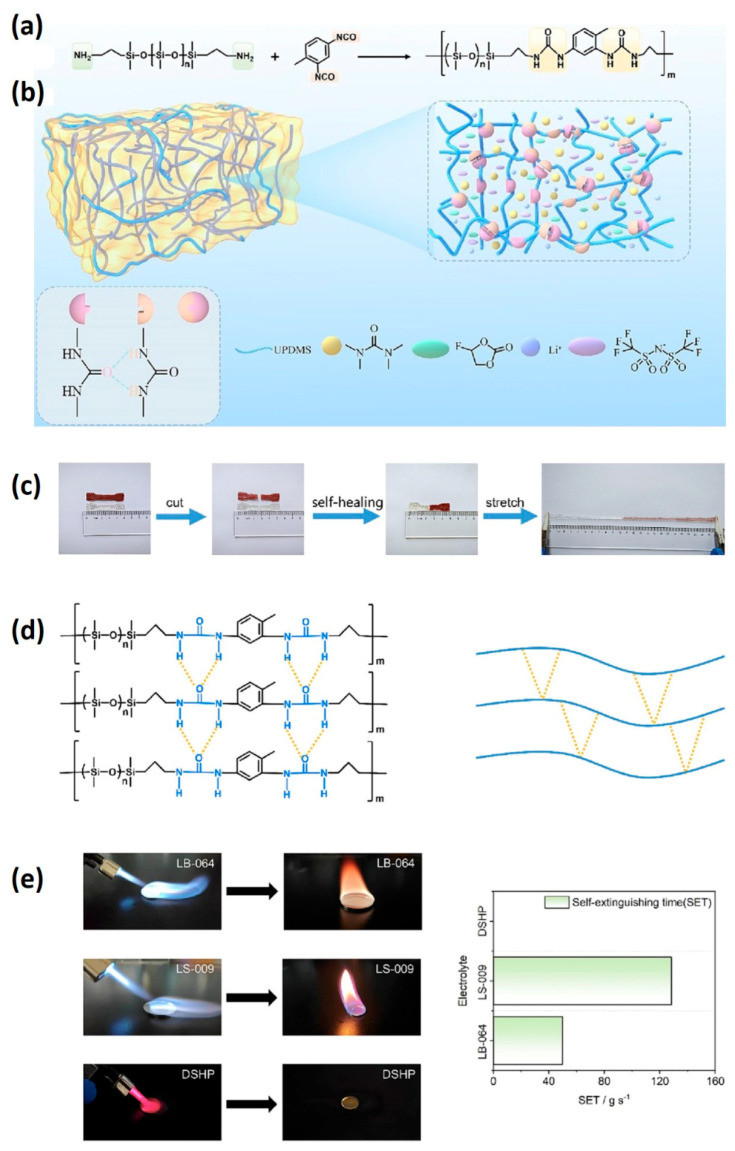
(**a**) Synthesis pathway of UPDMS chain; (**b**) schematic illustrating the design and mechanism of the DSHP gel electrolyte; (**c**) visual depiction of the DSHP self–healing and stretching tests; (**d**) schematic illustrating the self–healing mechanism of the DSHP electrolyte (highlighting main hydrogen bonds); (**e**) optical images showing ignition and SET tests of three samples (adapted with permission from Ref. [158], Copyright 2024 Elsevier.

Safety concerns surrounding lithium-ion batteries primarily stem from the utilization of flammable organic electrolytes, which pose a significant risk of fire and explosion due to their high heat potential. Non-flammable alternatives, such as deep eutectic solvents and ionic liquid-based electrolytes, have been explored to mitigate this hazard. However, polymer electrolytes, while non-flammable, often suffer from deformation and breakage during the charge–discharge process, leading to interface contact issues [154,155,156]. Addressing this challenge, self-healing electrolytes have emerged as a promising solution, capable of inhibiting such problems and improving electrochemical performance while reducing degradation. Polyethylene oxide (PEO)-based self-healing polymer electrolytes, for instance, introduce quadrupole hydrogen bonding but are limited by low ionic conductivity (2.1 × 10^−5^ S cm^−1^ at 30 °C) and low lithium-ion (Li^+^) migration numbers (0.38) [156,157]. In response to these limitations, the Chen group has developed a novel polydimethylsiloxane-based gel electrolyte (DSHP) incorporating a deep eutectic solvent (DES) [158]. This gel electrolyte effectively reduces side reactions between DES and lithium metal. Furthermore, the incorporation of fluoroethylene carbonate (FEC) as an additive decreases the viscosity of DES, facilitating the formation of solid electrolyte interphase (SEI) layers on the surface of the lithium metal anode (Figure 14).

The developed electrolyte exhibits non-flammability, an impressive ionic conductivity of 0.60 mS cm^−1^ at 30 °C, a high electrochemical voltage of 4.5 V vs Li/Li^+^, and a notable lithium-ion (Li^+^) migration number of 0.69. Moreover, cells employing this electrolyte configuration (Li|DSHP|Li) demonstrate exceptional cycle stability, lasting over 1400 h. When applied in quasi-solid-state lithium batteries with a lithium anode and LiFePO_4_ cathode, the DSHP electrolyte exhibits a high specific capacity of 151.6 mA h g^−1^, showcasing its potential for high-performance energy storage applications [158].

### 4.7. Nanogenerators Based on Triboelectric/Ultrasound

Triboelectric nanogenerators (TENGs), which harness mechanical energy from activities such as walking, talking, typing, and other motions, hold significant promise for powering various electronic devices. Among the potential candidates for stretchable and highly conductive hydrogels are ionic conductor hydrogels. These hydrogels, incorporating LiCl or NaCl into polyacrylamide matrices, exhibit notable characteristics, including high stretchability (ultimate strain ε_ult_ ∼ 2000%), softness, and biocompatibility. However, it is noteworthy that such hydrogels tend to dehydrate at temperatures above 30 °C and lose stability at relative humidity levels below 26% [159,160]. Addressing these challenges, the Mingzhe research group has developed a hydrophobic ionic liquid gel tailored specifically for TENG electrodes [161]. Comprising ethyl acrylate, polyethylene glycol diacrylate, and 1-butyl-2,3-dimethylimidazolium bis (trifluoromethyl sulfonyl) imide (HILG) on polydimethylsiloxane (PDMS), this innovative gel demonstrates exceptional properties, boasting stretchability of up to 400% and high transparency of 89%. Importantly, it maintains structural integrity for up to three months, even under extreme weather conditions ranging from −25 °C to 60 °C and relative humidity levels of up to 80%.

The Husam group employed MXene hydrogel as ultrasound energy harvesters, coupled with an implantable generator designed to convert ultrasound power into electric energy [162]. In this study, MXene, a two-dimensional material, and polyvinyl alcohol (PVA) were utilized to form a three-dimensional structure, leveraging the synergetic effects of bonding between MXene layers and the negatively charged surfaces of PVA. This structural arrangement not only facilitated the formation of a stable hydrogel but also imparted enhanced mechanical properties, attributed to MXene’s asymmetric strain sensitivity. The propagation of ultrasound waves induced coupling effects between acoustic and electric fields, leading to the generation of streaming vibration potentials within the MXene gel, thereby enhancing its energy harvesting capabilities. Furthermore, the MXene gel was augmented with triboelectrification, exhibiting promising potential for rapid charging of electric gadgets when implanted beneath a layer of beef approximately one centimeter thick. The Hong research group has developed a self-healable, anti-freezing, and biaxially stretchable triboelectric gel using amino- and hydroxy-terminated poly (dimethyl siloxane) (PDMS), isophorone diisocyanate, and silicone oil [163]. The resulting triboelectric nanogenerator device exhibits impressive performance metrics, including an open circuit voltage (VOC) of approximately 47 V, transferred charge (Qtr) of around 17 nC, and short-circuit current (ISC) of about 370 nA. Moreover, the device achieves a maximum power output of approximately 2000 μW m^−2^ and maintains stable performance even after 5000 cycles. The presence of reversible non-covalent bonds enables sustained energy harvesting potential through multiple cutting and self-healing processes, while also facilitating biaxial stretching of up to 150%.

### 4.8. Low Molecular Weight Gelator

The properties and functions of molecular gels are regulated by their gelation ability, thermal properties, mechanical strength, and stability. Low molecular weight gelators (LMWGs) are commonly employed to enhance the performance or introduce specific functionalities to gel assemblies. LMWGs, typically characterized by non-covalent interactions and having a molecular weight of less than 2000 Da, play a crucial role in the gelation process. Low molecular weight gelators (LMWGs) constitute a thermally reversible system that serves as renewable materials while also exhibiting considerable conductivity properties as a liquid electrolyte. These materials rely on non-covalent interactions, such as hydrogen bonding, electrostatic interactions, van der Waals forces, and π–π stacking, to form organic ionic gels. However, their utility is limited by the temperature range and mechanical properties within which they remain functional [164,165].

The Demchuk group utilized saccharine-based LMWGs, specifically methyl-4,6-O-(p-nitrobenzylidene)-α-D-glucopyranoside, for gelation alongside tetramethylammonium bromide as an electrolyte [166]. This resulted in the formation of a fibril-like network characterized by one-dimensional hydrogen bond chains in the solid state. Traditionally, polar solvents have been known to interfere with the gelation process by disrupting the gelator–gelator hydrogen bonds through electrostatic interactions. However, methyl-4,6-O-(p-nitrobenzylidene)-α-D-glucopyranoside possesses the unique property of being able to form a gel phase in both polar and non-polar solvents. The resulting organic ionic gel (OIG) exhibited conductivities of 121, 200, and 317 mS cm^−1^ at 25 °C, 50 °C, and 90 °C, respectively, when tested at a concentration of 3.7 M. The Kundu group designed a conjugated polymer, poly(3-hexylthiophene) (P3HT), in conjunction with low molecular weight gelators (LMWGs) such as di-Fmoc-L-lysine (di-Fmoc), to facilitate the formation of conductive gels [167]. Both components independently exhibit self-assembly, forming nanofibers in solution that maintain their structure in the solid phase upon drying. The resulting dried film contains 20% P3HT and exhibits electrical conductivity properties similar to pristine P3HT films (10^−6^–10^−9^ S cm^−1^). Molecular interactions between P3HT and LMWGs are minimal, with each component self-assembling independently while maintaining the integrity of the gel structure. This approach presents opportunities for the application of other conjugated polymers in the preparation of conductive gel formations. The Maruyama group fabricated a heterogeneous double-network ionogel using low molecular weight gelators (LMWGs), specifically 1,3,5-benzentricarboxylic acid and amino acid methyl esters, in combination with crosslinked polymers like PMMA [168]. LMWGs address the brittleness typically associated with ionogels while preserving their intrinsic ionic conductivity. In this system, LMWGs form the first network, while the crosslinked polymer forms the second network, resulting in the formation of 2nd network ionogels that maintain both mechanical strength and ionic conductivity. The ionogels exhibit high conductivities attributed to the large voids between entangled fibers, facilitated by the low gelator content, which allows for the movement of ions. Under compression stress, the double-network ionogels failed at a stress of 0.95 MPa, while PMMA ionogels failed at 0.34 MPa. These materials hold promise for use in energy devices.

**Table 1:** Performance comparison of gel materials for the different energy devices.

Devices	Materials	Solvent	Performances	Ref.
Mg–Air batteries	PEO/PAM hydrogel electrolyte	LiTFSi (acetone/dichloromethane)	Specific capacity: 2190 mAh g^−1^, Energy capacity: 2282 Wh kg^−1^	[126]
Li-ion batteries	3D LLTO hydrogel (PVA/GA)	LiTFSi (acetonitrile)	Li-ion conductivity: 8.8 × 10^−5^ S cm^−1^ at room temperature	[133]
OER	PA–PPY/CC	–	Over potential 340 mV at 10 mA cm^−2^, Cdl 1.85 mF cm^−2^, Tafel slope 54.9 mV dec^−1^, stability 20 h	[110]
ZABs	BFCs (PVA/PAN) catalyst	–	Capacity 691 mAh g^−1^ at energy density 798 Wh Kg^−1^ at –20 °C	[116]
Supercapacitors	PPH	Aqueous	Electrochemical capacitance of 306 mF cm^−2^ (153 F g^−1^), energy density of 13.6 Wh kg^−1^, tensile strength 5.3 MPa	[149]
ECDs	Polyacrylamide (PAAM) hydrogel	p-BQ/4–OH Tempo/HCl	Coloration efficiency of 291.35 cm^2^ C^−1^ for hydrogel–R and 152.18 cm^2^ C^−1^ for hydrogel–G, reversibility >200 cycles, short open time < 100 ms	[85]
ECDs	P[S-r-VBMI][PF6]–gel	[EMI][TFSI]	Elastic modulus 0.105 MPa	[84]
ECDs	10% (AG/P(AAm-AAc)-10/P2W18),	Aqueous	Optical contrast up to 65.54% at 700 nm	[86]
OER	Ni0.6Fe0.4–MOG	Nafion/ethanol/water	Over potential 289 mV at 10 mA cm^−2^, Tafel slope 33 mV dec^−1^	[91]
HER	Ni-Mo-P aerogel	–	69 mV at 10 mA cm^−2^	[92]
OER	Ni-Mo-P aerogel	–	Over potential 235 mV at 10 mA cm^−2^, water splitting efficiency cell voltage of 1.46 V at 10 mA cm^−2^	[92]
OER	Fe–Fe_2_O_3_/N-doped C	–	Onset potential 0.92 V, half-wave potential 0.72 V	[93]
OER	Ni (II)–triazole gel	–	Over potential 360 mV at 10 mA cm^−2^, Tafel slope 69 mV dec^−1^	[104]
HER	Ni (II)–triazole gel	–	Over potential 250 mV at 10 mA cm^−2^, Tafel slope 115 mV dec^−1^	[104]
HER	PNPN@MA hydrogel	H_2_PtCl_6_ solution	H_2_ production of 3.24 mmol h^−1^ g^−1^ under visible light irradiation	[107]
ZABs	TCOF-S-gel	tetramethylethylenediamine	ion conductivity of 27.2 mS cm^−1^ and a Zn^2+^ transference number of 0.89 Zn||TCOF-S-Gel||MnO_2_ discharge capacity 248 mAh g^−1^ at a 1C, after 1400 cycles polarization voltage 244 mV, redox potential difference 305 mV at the 1C	[115]
Stretchable conductive gels	PEDOT:PSS water/ethylene glycol organohydrogels	water/ethylene glycol	flexibility and strain sensitivity even at –40 °C, remodeling and self-healing properties	[135]

ECDs: electrochromic devices; C: carbon; N: nitrogen; OER: oxygen evolution reaction; ZABs: zinc–air batteries; HER: hydrogen evolution reaction; PAN: polyacrylonitrile; PVA: polyvinyl alcohol.

## 5. Summary and Outlook for Future Research

In future research endeavors, the multifunctional nature of conductive hydrogels must be carefully considered to enable their practical applications, encompassing stretchability, anti-freezing properties, self-healing capabilities, self-adhesion, and desirable electrochemical properties. Achieving a balance among different components is crucial for enhancing their overall performance and achieving a universal profile suitable for various applications. The construction of conductive gels relies on the incorporation of conductive polymers, metal elements, carbon-based materials, and ionic salts into 3D networks. However, maintaining stable operation poses challenges due to potential phase separation between conductive additives and polymer networks, resulting in mechanical and performance shortcomings.

Given the sensitivity of gel materials to environmental conditions, it is imperative to ensure their resilience to extreme temperatures and prolonged service periods. Overcoming challenges related to drying, freezing, and achieving self-healing properties is essential to maintaining integrity. For wearable applications, biocompatibility is paramount, especially when in close contact with the skin.

While fundamental research in lab settings is necessary, the technological readiness level for large-scale production or commercialization remains distant. Thus, the development of new technologies is essential to formulate products with novel characteristics. A fundamental understanding of formation processes and mechanistic characteristics is crucial for practical applications.

Mechanical properties, catalytic efficiency, and stability must be standardized to meet the requirements of various application areas. When incorporating carbon-based conductive fillers into gel materials, factors such as nanocrystallinity, degree of aromatic condensation, lateral size, and crystallite size must be considered to optimize conductivity effectively.

Design strategies based on in situ polymerization or post-polymerization methods can lead to the generation of cytotoxic oxidants and unreactive monomers. Therefore, special attention must be given, particularly in biomedical applications. Additionally, the evaluation of potential toxic hazards associated with carbon- or metal-based conductive fillers or supports is crucial for ensuring the safety of applications. Moreover, in the design of multifunctional conductive gels, there is currently no universally established protocol for defining the required properties. It is imperative to establish proper guidelines tailored to the specific multidimensional application areas to pave the way for future research exploration.

Overall, future research directions should focus on advancing the understanding of conductive hydrogel formation, enhancing their properties for specific applications, and overcoming existing challenges to enable widespread utilization in various fields.

## Data Availability

Not applicable.
